# Intravenous iron supplementation in heart failure patients induces temporary endothelial dysfunction with release of endothelial microvesicles

**DOI:** 10.3389/fimmu.2022.1092704

**Published:** 2023-01-24

**Authors:** Sebastian F. Mause, Martin Berger, Hwee Ying Lim, Felix Vogt, Vincent Brandenburg, Robert Stöhr

**Affiliations:** ^1^ Department of Internal Medicine I, Cardiology, University Hospital Aachen, RWTH Aachen University, Aachen, Germany; ^2^ Immunology Translational Research Programme, Yong Loo Lin School of Medicine, Department of Microbiology & Immunology, National University of Singapore, Singapore, Singapore; ^3^ Immunology Programme, Life Sciences Institute, National University of Singapore, Singapore, Singapore; ^4^ Department of Cardiology and Nephrology, Rhein-Maas Klinikum, Wuerselen, Germany

**Keywords:** extracellular vesicles, microvesicles, microparticles, endothelial dysfunction, heart failure, cardiovascular disease, iron, ferric carboxymaltose

## Abstract

**Background:**

Intravenous iron supplementation is an established therapy for patients with heart failure (HF) and concomitant iron deficiency reducing the risk of HF hospitalization. However, concerns persist regarding potential adverse vascular effects, since iron may induce oxidative stress, inflammation, and apoptosis of endothelial cells. To assess endothelial health following ferric carboxymaltose (FCM) administration, we analyzed the profile of circulating endothelial microvesicles (EMVs) and endothelial progenitor cells (EPCs) in a cohort of 23 HF patients using flow cytometry.

**Results:**

Compared to healthy subjects, baseline levels of CD31+/CD41- EMVs were higher and EMVs featured a more apoptotic phenotype in HF patients. Following FCM administration, EMV levels showed a rapid but transient increase and displayed an altered phenotype profile with dominant augmentation of EMVs expressing inducible markers CD62E and CD54, indicating endothelial inflammatory activation and injury. Levels of circulating vasoregenerative CD45lowCD34+KDR+ EPCs were lower in HF patients and FCM application resulted in an early decrease of EPCs followed by substantial mobilization into the circulation after one week. Levels of EMVs and EPCs returned to baseline values within two and four weeks, respectively. HF patients with additional chronic kidney disease showed an elevated EMV/EPC ratio and diminished EPC mobilization, suggesting impaired vascular repair capacity. Providing a mechanistic link, *in vitro* experiments with cultured endothelial cells revealed that FCM dose-dependently promotes endothelial apoptosis, increases expression of adhesion molecules and CXCL12, and triggers generation of EMVs.

**Conclusion:**

Intravenous iron supplementation with FCM in HF patients induces a biphasic response with initial increased release of CD62E+ and CD54+ enriched EMVs and subsequent mobilization of EPCs, indicating endothelial dysfunction upon FCM and suggesting consecutive engagement of a defense program aimed to reconstitute vascular health.

## 1 Introduction

Iron deficiency is a prevalent comorbidity in patients with heart failure (HF), related to exercise intolerance and independently predicting poor outcome in chronic and acute settings (1, 2). Randomized controlled trials have shown that iron supplementation with intravenous ferric carboxymaltose (FCM) improves symptoms, quality of life and exercise capacity in iron deficient heart failure patients. Recently, the prospectively designed AFFIRM-AHF trial demonstrated that administration of FCM reduced the composite endpoint of first HF hospitalization or cardiovascular death and total HF hospitalizations in HF patients with concomitant iron deficiency (3). In light of this evidence, the current 2021 guidelines of the European Society of Cardiology confirmed a class IIa indication for iron supplementation with FCM in symptomatic, iron-deficient patients with reduced left ventricular ejection fraction (≤50%) to limit the risk of HF hospitalization (4). Of note, evidence for a beneficial effect on all-cause and cardiovascular mortality in iron-deficient heart failure patients is lacking so far (5); however, ongoing trials are expected to provide more data on the effects of FCM and other iron formulations in a wider spectrum of HF patients (2).

Considering the potential of iron formulations to induce oxidative stress and inflammation, some concerns persist regarding the long-term safety of intravenous iron supplementation, the comparison of different iron formulations and the clinical outcome in various patient populations (6, 7). So far, experimental and clinical data suggest that iron administration may induce inflammatory activation and apoptosis of vascular cells and finally lead to functional disturbances of the endothelium through reactive oxygen species and lipid peroxidation (8–11). Excessive iron therapy as well as iron overload may exacerbate atherosclerosis by iron-triggered lipid profile alterations and ferroptosis, stimulate release of pro-atherogenic inflammatory mediators, and impair endothelial homeostasis with altered vascular permeability (7, 12). FCM as a stable complex with a carboxymaltose shell enabling gradual release of iron predominantly in the reticuloendothelial system is thought to be associated with a relatively lower risk of oxidative stress compared to less stable iron formulations, however data supporting a clear benefit of FCM is conflicting so far and clinical evidence is scarce (10, 13).

Circulating endothelial microvesicles (EMVs, also termed endothelial microparticles or endothelial-derived extracellular vesicles) have emerged as a biomarker and effector of endothelial dysfunction as well as a predictor of future cardiovascular events in patients with coronary artery disease and HF (14–18). The formation of such membrane-enclosed vesicles that are released upon cellular activation or apoptosis is a selective process and contextual cues crucially determine their configuration and composition. Consequently, EMVs and their phenotype may reflect the current state of the parental endothelial cells, rendering them as attractive indicator of vascular health (15). The mobilization and homing of endothelial progenitor cells (EPCs) can be regularly found in response to endothelial stress and injury (19). Due to their regenerative properties and their potential to maintain and restore endothelial integrity, EPCs have emerged as a surrogate marker for the intrinsic vascular repair capacity and may together with EMVs reflect endothelial homeostasis (20–22).

Given the evidence that alteration in endothelial homeostasis is a common feature in HF patients and in light of potential deleterious vascular effects of iron supplementation, we now determined in a cohort of HF patients the levels of circulating EMVs and EPCs to monitor endothelial health following FCM administration.

## 2 Materials and methods

### 2.1 Patients and control subjects

We enrolled 23 subjects (mean age 67 years) from the IRON-TURTLE trial, a single-center prospective interventional pilot study which included symptomatic, hospitalized heart failure (HF) patients with reduced ejection fraction (HFrEF, LV-EF ≤ 40%) and concomitant iron deficiency receiving a single dose of 1000 mg ferric carboxymaltose intravenously (FCM; Ferinject, Vifor Pharma, Munich, Germany). A summary of patient characteristics is shown in **Table 1**. In accordance with the current guidelines of the European Society of Cardiology (ESC; 4), iron deficiency in HF patients was defined as either a serum ferritin concentration <100 ng/mL or 100-299 ng/mL with transferrin saturation (TSAT) <20%. 16 (70%) of the 23 patients with HFrEF had an ischemic cardiomyopathy, defined as presence of history of myocardial infarction, coronary revascularization, or obstructive coronary artery disease (>50% stenosis). All HF patients received optimal medical therapy in accordance with the current ESC heart failure guidelines for at least 2 months (4). Inclusion criteria were age from 18 to 85 years, signed written informed consent, LV-EF ≤ 40% as documented by echocardiography or cardiac MRI not older than 3 months before study inclusion. Major exclusion criteria were acute myocardial infarction, stroke, or surgery in the last 30 days, clinical or biochemical evidence for the presence of concomitant inflammatory disease, relevant arrythmia, advanced chronic kidney disease (CKD; eGFR < 15 ml/min/1.73 m^2^), relevant liver dysfunction, autoimmune or malignant disease, thrombocytopenia (<100 000/L), anemia (hemoglobin <8.5 g/dL), pregnancy, inability to understand the consent form, participation in or consent to participate in another study. Based on the presence or absence of chronic kidney disease (CKD), patients were divided into two groups with a cut-off eGFR of 60 ml/min/1.73 m^2^ (non-CKD group n = 11, CKD-group n = 12). 15 healthy subjects aged 40 to 72 years (mean age 62 years) without any evidence of HF by history and physical examination served as a control group. All study participants gave written informed consent, and the study was approved by the ethics committee of RWTH Aachen University (Aachen, Germany).

**Table 1:** Summary of patient characteristics.

	All HF patients (n=23)	non-CKD HF (n=11)	CKD HF (n=12)	p-value
Age (years±SD)	67±12	61±11	72±11	0.027
Male/Female	12/11	6/5	6/6	0.827
Cardiovascular Risk Factors				
Smoking	5 (22%)	4 (36%)	1 (8%)	0.103
Diabetes mellitus	11 (47%)	4 (36%)	7 (58%)	0.292
Arterial Hypertension	16 (70%)	7 (64%)	9 (75%)	0.554
Hyperlipidemia	13 (56%)	6 (55%)	7 (58%)	0.855
Obesity	8 (35%)	4 (36%)	4 (33%)	0.879
BMI	27.7±7.8	27.1±7.4	28.3±8.6	0.898
HF Characteristics								
ICM HFrEF	16 (70%)	7 (64%)	9 (75%)	0.554
Non-ICM HFrEF	7 (30%)	4 (36%)	3 (25%)	0.554
NYHA I	0	0	0	
NYHA II	13 (57%)	6 (55%)	7 (58%)	0.855
NYHA III	10 (43%)	5 (45%)	5 (42%)	0.855
NYHA IV	0	0	0	
Kidney Function								
S-Creatinin (mg/dl)	1.61	0.99	2.18	<0.0001
eGFR (ml/min/m2)	50	75	28.2	<0.0001
Cystatin C (mg/l)	1.85±0.7	1.15±0.42	2.49±0.48	<0.0001
Iron Metabolism								
Ferritin (μg/l)	67±48	77±53	59±42	0.525
Transferrin (g/l)	297±47	303±30	291±59	0.786
Iron (μmol/l)	64±33	77±40	51±21	0.069
Soluble transferrin receptor (mg/l)	5.6±3.3	3.8±1.9	7.2±3.4	0.008
Transferrin saturation (%)	15.8±8.7	18.8±10.4	13±6	0.138
Inflammation and Others								
hsCRP (mg/l)	6.8±6.1	5,3±6.3	8.1±6.9	0.275
IL6 (mg/dl)	15.9±12.1	16.9±15.4	15.2±10.3	0.976
NT-proBNP (ng/l)	3947±2219	4927±2816	4459±3210	0.048
AST (U/l)	29.3±13.2	34.6±14.5	24.4±10.1	0.100
HF Medication								
ACE-I/ARB	16 (70%)	7 (64%)	9 (75%)	0.554
ARNI	6 (26%)	3 (27%)	2 (17%)	0.538
β-blockers	22 (96%)	11 (100%)	11 (91%)	0.328
MRA	11 (48%)	6 (55%)	5 (42%)	0.879
Diuretic	23 (100%)	11 (100%)	12 (100%)	0.999
Statin	16 (70%)	7 (64%)	9 (75%)	0.554

### 2.2 Isolation and flow cytometry-based analysis of microvesicles

To isolate MVs from human subjects, blood was drawn using a 20-gauge needle or larger and collected in tubes containing sodium citrate at a final concentration of 0.106 mol/L (Sarstedt, Nümbrecht, Germany). Prolonged use of a tourniquet was avoided and the first 2.7 mL of collected blood was not used for MV analysis. In general, blood was collected in the morning, except for samples collected 3 hours after FCM administration. Differential centrifugations were done in accordance with current guidelines (23). Briefly, platelet-free plasma (PFP) was obtained after two sequential centrifugations at 2,500 g for 15 min (21°C, no brake, swinging bucket rotors). The obtained supernatant containing MVs was subsequently subjected to incubation (in 50 µL aliquots) for 30 min at room temperature with directly conjugated antibodies for identification of specific cell surface markers and characterization of MVs. We used the following mouse anti-human mAbs (all BioLegend, San Diego, CA, USA): FITC-conjugated CD41 mAb (clone HIP8), APC-conjugated CD31 mAb (clone WM59), PE-conjugated CD144 mAb (clone BV9), PerCP/Cyanine5.5-conjugated CD54 mAb (clone HA58), PE-Cy7-conjugated CD62E mAb (clone HAE-1f), PE-conjugated CD45 mAb (clone HI30), and PerCP/Cyanine5.5-conjugated CD235a (Glycophorin A) mAb (clone HI264). Antibodies were at saturating concentrations as determined by preceding titration experiments. Isotype-matched IgG1 or IgG2a (all BioLegend) were used as a negative control and applied to identify nonspecific staining. For polychromatic flow cytometric analysis, we additionally used fluorescence minus-one (FMO) controls to define gates. All antibodies were subjected to centrifugation at 20,000 g for 7 min prior to use for removal of antibody aggregates. Following staining, all samples were diluted to 1.5 ml with double-filtered PBS (0.22 μm membrane filter; Millipore, Darmstadt, Germany) and analyzed with a BD FACSCanto II flow cytometer (BD Biosciences, Franklin Lakes, NJ, USA) at the low flow-rate setting and a run time of 180 sec per sample. Application of size standard beads with a diameter of 0.3, 0.5 and 1.0 μm (Nanobead and Microbead NIST Traceable Particle Size Standards, Polysciences, Warrington, PA, USA) together with analysis of non-activated and activated platelets as a reference allowed estimation of size distribution and gating of the MV population. TruCount beads (BD Biosciences) with a known number of calibrated and fluorescent 4.2 μm microbeads were used for quantification of MVs following the manufacturer’s instructions and absolute MV counts were noted as events per μl. Generally, MVs were defined as events ≤ 1.0 μm in diameter and positive for cell-specific markers. Platelet-derived MVs (PMVs) were defined as CD41+ size-gated events (≤ 1.0 μm in diameter), endothelial MVs (EMVs) as CD31+/CD41- or CD144+/CD41- size-gated events, leucocyte-derived MVs (LMVs) as CD45+ size-gated events and erythrocyte-derived MVs as CD235a+ size-gated events. To confirm the vesicular origin of the preparation, PFP was depleted from MV by sample lysis with 0.5% Triton buffer (Triton X-100, Sigma-Aldrich, Darmstadt, Germany). To address swarm detection, we performed in preliminary experiments serial dilutions (1:10, 1:20, 1:100, and 1:200) of the obtained PFP. There was no relevant change in MV counts across dilutions in the analyzed PFP. As a quality control, reagent blanks and sample blanks were analyzed to ensure that low counts were maintained and to evaluate background auto-fluorescence counts. The above mentioned measures and procedures ensured limitation of inadequate detection of artefacts affecting enumeration of circulation MVs. Scanning electron microscopy (ScEM; FEI/Philips ESEM XL30 FEG, Amsterdam, Netherlands), performed as previously described (24), confirmed the presence of MVs and revealed that the majority of MVs measured 200 to 800 nm. As assessed by flow cytometric size profiling and scanning electron microscopy, we did not observe a substantial proportion of apoptic bodies as extracelluar vesicles sized >1.0 µm.

### 2.3 Detection of EPCs using flow cytometry

Quantitative analysis of CD45lowCD34+KDR+ EPCs using flow cytometry was performed according to established protocols with a lyse/no-wash procedure, sequential gating and dead cell exclusion (25). For detection of viable EPCs, we used 7-AAD (BD Biosciences) and the following mouse anti-human mAbs (all BD Biosciences): FITC-conjugated CD45 mAb (clone 2D1), APC-conjugated CD34 mAb (clone 8G12), and PE-conjugated KDR mAb (CD309/VEGFR2). For the FMO control we used an equivalent amount of the labeled anti-CD45 and anti-CD34 mAb. The mAbs and 7-AAD were pipetted into Trucount tubes (BD Biosciences) together with 50 μL of patient samples and incubated for 20 min at room temperature protected from light. Red blood cells were lysed by adding 450 μl of 1x lysing solution (BD Biosciences) to each tube and incubated for 10 min at room temperature in the dark. Subsequently, tubes were placed immediately on ice until ready to perform flow cytometry analysis. Applying sequential gating (25), EPCs were finally counted as CD45lowCD34+KDR+ and 7-AAD negative cells in the lymph-blast scatter region.

### 2.4 Culture and analysis of HUVECs

Culture of human umbilical vein endothelial cells (HUVECs; PromoCell, Heidelberg, Germany) was performed in accordance to established protocols (26). HUVECs were grown to confluence in endothelial cell growth medium (PromoCell) and used at passage 3 to 5. For experiments analyzing the role of FCM, cells were incubated at 37°C in the absence (control) or presence of FCM at concentrations ranging from 0.1 to 0.4 mg/mL, which in a 70-kg individual corresponds to a pharmacological application of ∼400, ∼800 and ∼1600 mg iron. Apoptosis of HUVECs, left untreated or treated with various doses of FCM for 24 hours, was measured using the Apoptosis Detection kit I (BD Biosciences) per manufacturer’s recommendations and performing flow cytometry. As a standard, 1 × 10^5^/mL of cells per treatment condition were fixed and stained with 5 μL Annexin V–FITC and 5 μL propidium iodide (PI) for 20 minutes. Early apoptotic cells were defined as PI negative and FITC Annexin V positive cells, late apoptotic/necrotic cells were defined as PI and FITC Annexin V positive cells.

Flow cytometry was employed to determine the expression of the adhesion molecules CD62E (E-selectin) and CD106 (VCAM-1) as well as the chemokine CXCL12 on the surface of HUVECs. HUVEC monolayers, left untreated or treated withvarious doses of FCM for 24 hours, were briefly washed with PBScontaining 0.1% BSA and fixed with 1% formaldehyde (Sigma-Aldrich) in PBS at 4°C for 5 min. Fixation was stopped by diluting the fixative with PBS (1:10) and washing the cells with PBS containing 0.1% BSA. HUVEC monolayers were subsequently incubated with PE-conjugated mouse anti-human CD62E (BioLegend), APC-conjugated mouse anti-human CD106 (BioLegend) or with FITC-conjugated mouse anti-human CXCL12 (R&D Systems, Minneapolis, MN, USA) at room temperature for 30 minutes. Isotype-matched IgG1 or IgG2a used as a negative control were purchased from the same manufacturer as the immune Abs. Following washing with PBS containing 0.1% BSA, HUVEC monolayers were treated with trypsin/EDTA (0.025%) and subsequently washed with ice-cold PBS containing 5% FCS. Finally, HUVECs were carefully scraped, separated by repeated pipetting, and after washing with PBS transferred to flow cytometry tubes for subsequent analysis.

To analyze and quantify the generation of EMVs by HUVECs, the supernatant of HUVEC monolayers left untreated or treated with various doses of FCM for up to 48 hours was collected and subjected to sequential centrifugation performed twice at 2500 g for 10 min for removal of cell debris. Subsequent flow cytometry analysis of HUVEC-derived EMVs followed the protocol as described above for patient MV characterization. HUVEC-derived EMVs were defined as events ≤ 1.0 μm in diameter and positive for CD31.

### 2.5 Statistical analysis

Data was analyzed for normality using Shapiro–Wilk test. For non-normally distributed data, we used the non-parametric Mann–Whitney test and Kruskal–Wallis test with *post-hoc* Dunn test for unmatched pairs and Wilcoxon and Friedman test with *post-hoc* Dunn test for matched pairs using the GraphPad Prism version 8 (GraphPad Software, San Diego, CA, USA) as appropriate. For normally distributed data, we applied the Student’s t-test and one-way ANOVA followed by Newman–Keuls *post-hoc* test. Correlation analysis was performed using Spearman’s correlation coefficient. Differences with p < 0.05 were considered to be statistically significant. The number of independent experiments is stated in the figure legends.

## 3 Results

### 3.1 FCM administration induces temporary increase in circulating EMV levels

**Figure 1:**
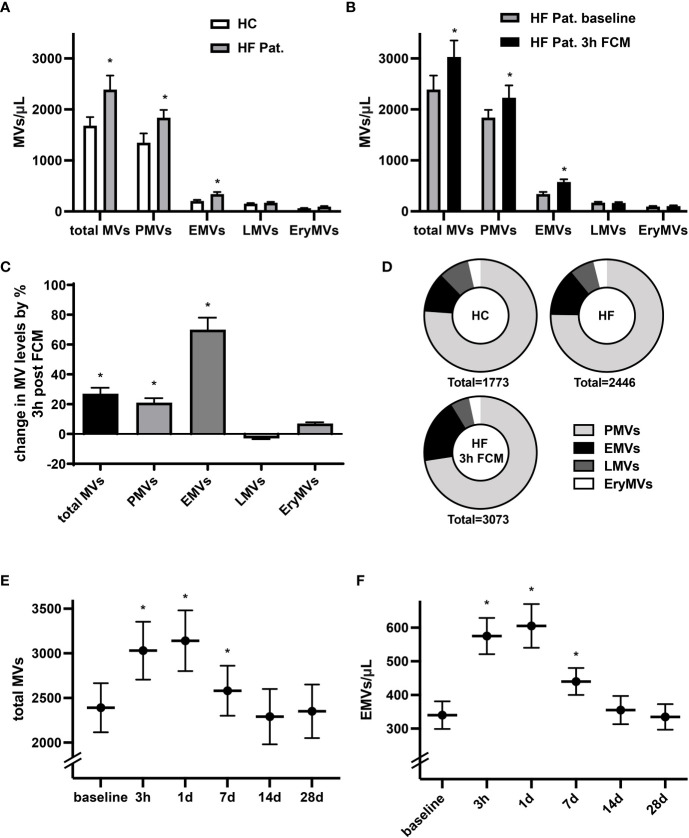
Administration of FCM in HF patients induces temporary increase in MV concentration. **(A)** Levels of circulating MVs and their subpopulations in HF patients with reduced LV-function and iron deficiency compared to healthy controls (HC). MVs and their phenotypic profile were characterized by flow cytometry and identified as CD41+ platelet-derived MVs (PMVs), CD31+/CD41- endothelial MVs (EMVs), CD45+ leucocyte-derived MVs (LMVs), and CD235a+ erythrocyte MVs (EryMVs). *p < 0.05 vs. healthy controls for the respective MV population. **(B)** Levels of circulating MVs and their subpopulations in HF patients at baseline and 3 hours after FCM application. *p < 0.05 vs. baseline for the respective MV population. **(C)** Relative changes in MV levels three hours after FCM application compared to baseline. *p < 0.05 vs. baseline **(D)** Relative share of various MV subpopulation in healthy controls and HF patients as indicated. **(E, F)** Time course analysis of MV and EMV levels following FCM injection in HF patients. *p < 0.05 vs. baseline for the respective MV population.

Levels of circulating MVs are thought to indicate not only cellular activation and apoptosis but may also denote pathophysiological conditions. In line with previous reports (27), we identified significantly higher baseline levels of circulating total MVs, CD41+ platelet-derived MVs (PMVs) and CD31+/CD41- EMVs in HF patients with reduced LV-function and iron deficiency compared to healthy controls (**Figure 1A**). In HF patients, levels of circulating total EVs, PMVs and CD31+/CD41- EMVs were considerably higher three hours after intravenous administration of 1000 mg ferric carboxymaltose (FCM) compared to baseline (**Figures 1B, C**). Among the MV subpopulations, FCM-induced alterations in levels of circulating MVs were most dominant for EMVs with a relative increase of 70%, whereas levels of total MVs rose by only 27% (**Figure 1C**). Accordingly, supplementation of FCM mediated a shift in the relative contribution of EMVs to total MVs from 14 to 19% (**Figure 1D**). Leukocyte-derived CD45+ MVs (LMVs) and erythrocyte-derived CD235a+ MVs (EryMVs) represented only a minority of all MVs (9.1% and 4.1% of total MVs, respectively) and levels of these subpopulations did not significantly differ among the three different groups (**Figures 1A–C**). Assessing MV release over time, we found that levels of total MVs and EMVs peaked at day one and returned to baseline levels within 14 days following FCM application (**Figures 1E, F**).

**Figure 2:**
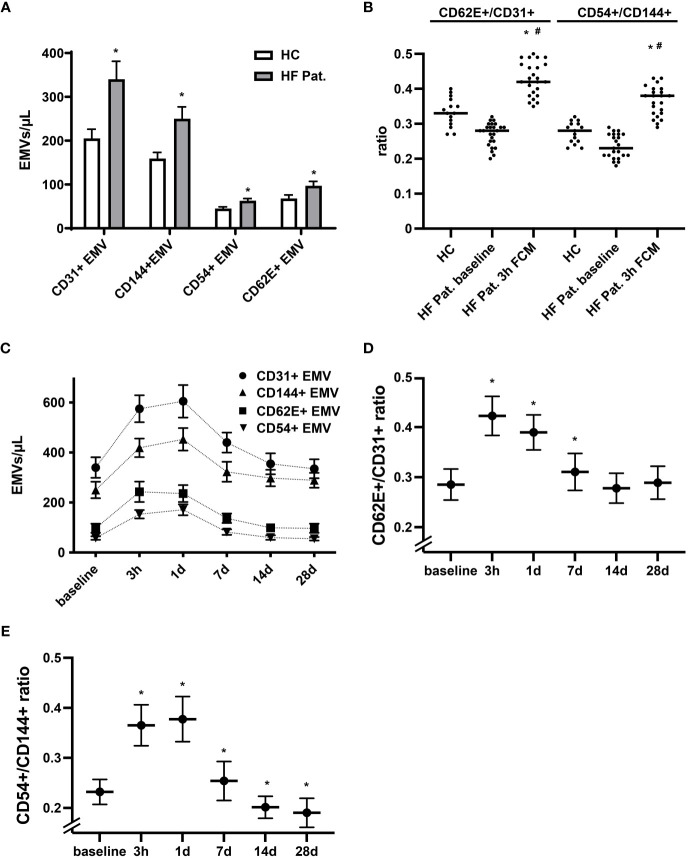
Dynamic alterations in the phenotype of circulating EMVs following FCM application. **(A)** Analysis of the phenotype of circulating EMVs with assessment of the constitutive endothelial markers CD31 (PECAM-1) and CD144 (VE-Cadherin) and the inducible markers CD62E (E-selectin) and CD54 (ICAM-1). *p < 0.05 vs. HF patients at baseline for the respective EMV phenotype. **(B)** Evaluation of the EMV CD62E+/CD31+ and CD54+/CD144+ ratio to determine the balance of activation vs. apoptosis related generation of EMVs. *p < 0.05 vs. the respective ratio in healthy controls, ^#^p < 0.05 vs. the respective ratio in HF patients at baseline. **(C)** Kinetics of various EMV phenotypes following FCM injection in HF patients. Data points for each phenotype marker are connected by a broken line for the sole reason to enhance readability **(D, E)** Kinetics of the EMV CD62E+/CD31+ and CD54+/CD144+ ratio at baseline and following FCM injection in HF patients. *p < 0.05 vs. baseline for the respective EMV ratio.

The pattern of membrane proteins expressed by EMVs is related to the process and context that triggers the release of MVs. In accordance with this concept, EMVs generated during activation display a different phenotype than EMVs released during apoptosis (28, 29). EMVs expressing constitutive markers of endothelial cells, such as CD31 (PECAM-1), and CD144 (VE-Cadherin) are more typically found in apoptosis, while those expressing inducible markers such as CD54 (ICAM-1) and CD62E (E-selectin) are increased dominantly during activation and inflammation of the endothelium (27, 29). Performing phenotypic profiling of circulating EMVs, we found in HF patients at baseline not only higher levels of CD31+ EMVs, but also augmented levels of EMVs expressing CD144, CD62E and CD54 when compared to healthy controls (**Figure 2A**). Generally, we detected a trend towards a lower CD62E+/CD31+ and CD54+/CD144+ EMV ratio in the HF cohort, suggestive of a higher degree of endothelial apoptosis (**Figure 2B**). In contrast, administration of FCM in HF patients resulted in a dynamic, significant, and reversible phenotype shift of EMVs. Three hours after FCM supplementation, we registered a dominant rise of CD62E+ EMVs and CD54+ EMVs together with a corresponding increase in the EMV CD62E+/CD31+ and CD54+/CD144+ ratio, indicating intensified activation of the endothelium (**Figures 2B–E**). After 14 days, the relative dominance of CD62E+ and CD54+ expressing EMVs vanished and the CD62E+/CD31+ and CD54+/CD144+ ratio returned to or even dropped below baseline values, reflecting a switch towards a more apoptotic EMV phenotype (**Figures 2D, E**).

### 3.2 Mobilization of circulating endothelial progenitor cells following FCM application

**Figure 3:**
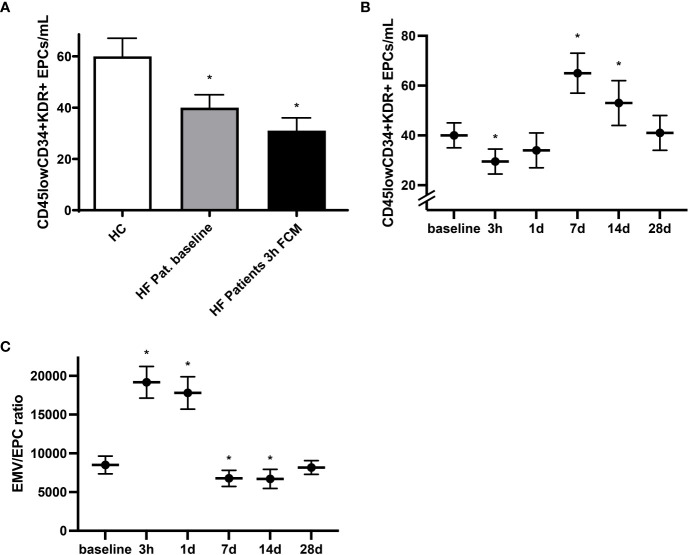
Mobilization of circulating EPCs following FCM application. **(A)** Levels of circulating CD45^low^CD34^+^KDR^+^ EPCs in healthy controls and HF patients at baseline and three hours after FCM application as assessed by flow cytometry. *p < 0.05 vs. healthy controls. **(B)** Time course analysis of CD45^low^CD34^+^KDR^+^ EPCs in HF patients at baseline and following FCM exposure. *p < 0.05 vs. baseline. **(C)** Time course analysis of EMV/EPC ratio in HF patients. *p < 0.05 vs. baseline.

In light of the regenerative potential of EPCs as well as their mobilization following vascular injury and involvement in endothelial repair, identification of circulating EPCs may, together with assessment of EMVs, constitute an integrative marker of vascular health (15, 19). At baseline we detected lower levels of circulating CD45^low^CD34^+^KDR^+^ EPCs in HF patients compared to healthy individuals (**Figure 3A**). Time course analysis revealed that in response to iron administration, levels of circulating CD45^low^CD34^+^KDR^+^ EPCs dropped by roughly 30% within the first three hours before reaching even substantially higher levels compared to baseline at day seven and day 14 (**Figures 3A, B**). We next assessed the ratio of EMVs to EPCs as a measure of the balance between endothelial activation and endothelial repair capacity (30, 31). Compared to healthy controls, the EMV/EPC ratio was elevated in HF patients at baseline (data not shown). Following exposure to FCM, we detected a drastic but temporary increase in the EMV/EPC ratio, highlighting the biphasic sequence of initial endothelial activation with subsequent and delayed EPC mobilization (**Figure 3C**). Mainly driven by the marked increase of circulating EPCs seven days after FCM application, the EMV/EPC ratio finally dropped below the baseline value and normalized within 28 days.

### 3.3 Aggravated endothelial dysfunction in HF patients with chronic kidney disease

**Figure 4:**
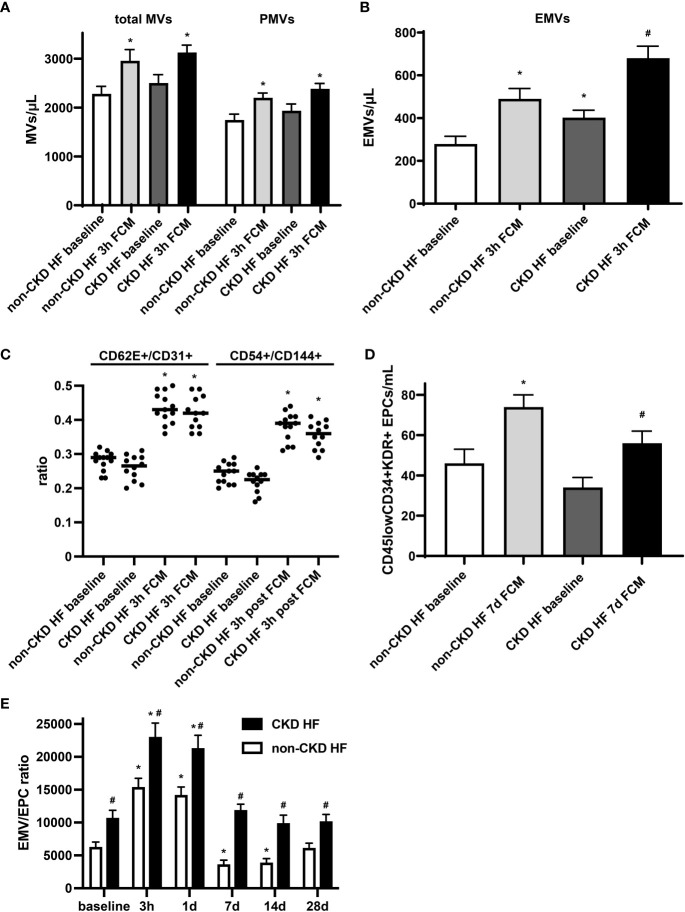
Circulating EMVs and EPCs in HF patients with CKD. **(A, B)** Levels of circulating MVs and their subpopulations in HF patients with or without additional CKD at baseline and three hours after FCM application. *p < 0.05 vs. non-CKD HF patients at baseline, #p < 0.05 vs. non-CKD HF patients three hours after FCM application. **(C)** EMV CD62E+/CD31+ and CD54+/CD144+ ratio in HF patients with/without CKD. *p < 0.05 vs. the respective ratio at baseline. **(D)** Assessment of CD45^low^CD34^+^KDR^+^ EPCs in HF patients with/without CKD at baseline and seven days after FCM application as noted. *p < 0.05 vs. non-CKD HF patients at baseline, ^#^p < 0.05 vs. non-CKD HF patients seven days after FCM application. **(E)** Time course analysis of EMV/EPC ratio in HF patients with CKD. p < 0.05 vs. the respective ratio at baseline. #p < 0.05 vs. non-CKD HF patients.

Endothelial dysfunction is evident at early stages in patients with chronic kidney disease (CKD), and its prevalence is incrementally higher as the disease progresses toward end stage renal disease (32). In a subgroup analysis of HF patients, we observed that in the CKD group, levels of total MVs and PMVs were moderately and levels EMVs were significantly higher at baseline compared to the non-CKD group. EMVs were found to be negatively related to eGFR in HF patients with a Spearman correlation coefficient of -0.84 (p <0.0001). Furthermore, EMVs showed a more pronounced increase in absolute numbers following iron exposure, confirming the link between chronic kidney disease and endothelial dysfunction (**Figures 4A, B**). The EMV phenotype and its changes following FCM supplementation did not significantly differ between CKD and non-CKD HF patients (**Figure 4C**). In contrast, levels of circulating CD45^low^CD34^+^KDR^+^ EPCs in the CKD subgroup were lower before and after FCM application and the degree of EPCs mobilization seven days after iron exposure was dampened compared to the non-CKD group (**Figure 4D**). Consequently, the EMV/EPC ratio was substantially higher throughout all assessed time points, suggesting an impaired endothelial repair capacity in CKD HF patients (**Figure 4E**).

### 3.4 FCM induces apoptosis and inflammatory activation of cultured HUVECs and triggers enhanced release of EMVs

**Figure 5:**
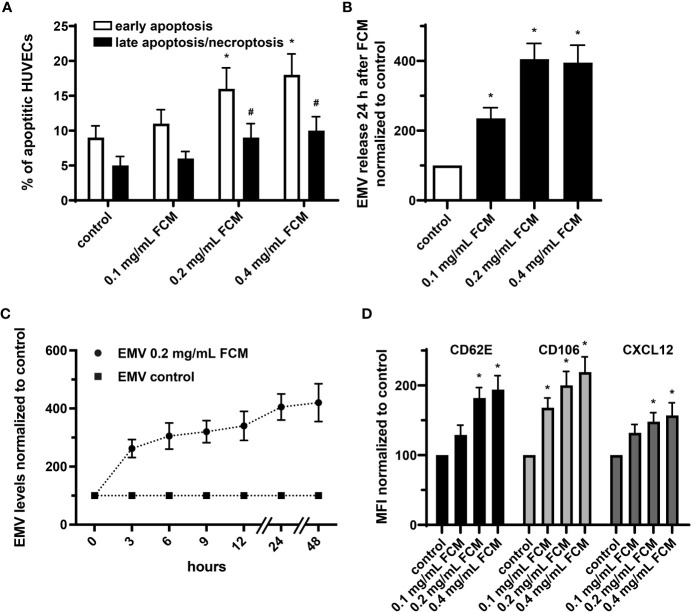
FCM exposure induces apoptosis and inflammatory activation of HUVECs and promotes release of EMVs. **(A)** Annexin V-FITC/PI staining with subsequent flow cytometry analysis to determine the rate of apoptotic HUVECs treated as indicated for 24 hours. *p < 0.05 vs. untreated HUVECs (control) for early apoptosis, ^#^p < 0.05 vs. control for late apoptosis/necroptosis; n = 5. **(B)** Analysis of EMV release by HUVECs treated as indicated for 24 hours. Levels of HUVEC-derived EMVs were normalized to untreated HUVECs (control). *p < 0.05 vs. untreated HUVECs (control); n = 6. **(C)** Time course analysis of EMV release by HUVECs kept untreated or treated with 0.2 mg/mL FCM for indicated time points. Levels of EMVs derived from FCM-treated HUVECs were normalized to levels of EMVs derived from untreated HUVECs for each time point (control); n=5. **(D)** Flow cytometry-based assessment of CD62E, CD106 and CXCL12 on the surface of HUVECs treated as indicated. Data are expressed as mean fluorescence intensity (MFI) in % normalized to untreated HUVECs (control). *p < 0.05 vs. untreated HUVECs (control) for the respective marker; n = 5.

It has been shown that endothelial cells treated with iron sucrose showed altered morphological characteristics with the presence of cellular fragmentation and increased apoptosis. We now applied FCM to HUVECs at concentrations ranging from 0.1 to 0.4 mg/mL, which in a 70-kg individual corresponds to a pharmacological application of ∼400, ∼800 and ∼1600 mg iron. Such exposure to FCM resulted in dose-dependent changes in cell viability with an increase of early apoptosis and late apoptosis/necroptosis in the presence of higher iron doses (**Figure 5A**). Furthermore, administration of FCM dose- and time-dependently induced augmentation of EMV shedding by HUVECs and triggered increased endothelial expression of CD62E (E-selectin) and CD106 (VCAM-1), indicating inflammatory activation of cultured endothelial cells in response to FCM (**Figures 5B–D**). In light of evidence that the chemokine CXCL12 takes center stage in the vascular recruitment of progenitor cells including EPCs, we also observed elevated expression of CXCL12 by HUVECs following exposure to FCM (**Figure 5D**).

## 4 Discussion

Iron supplementation with intravenous FCM has been shown to relieve symptoms in HF patients with iron deficiency and is nowadays an established complimentary therapy limiting the risk of HF hospitalization (4). Acknowledging the potential of iron to promote oxidative stress and vascular inflammation and considering concerns regarding iron-induced endothelial disturbances, we now sought to assess endothelial health and homeostasis following FCM administration in HF patients by analyzing temporal alterations in the profile of circulating EMVs and EPCs. Collectively, our data now reveal that intravenous iron supplementation with FCM results in early endothelial dysfunction with increased release of CD62E- and CD54-expressing EMVs and consecutive promotion of vascular repair mediated by mobilization of regenerative EPCs.

Several studies demonstrated that enhanced generation and release of MVs from the endothelium is a hallmark event indicating the onset of endothelial activation and dysfunction (15, 33, 34), thus rendering EMVs as an attractive surrogate marker of endothelial health. Data from clinical studies suggested that endothelial dysfunction is a common feature for the propagation and development of HF beyond etiology and elevated levels of circulating EMVs have been identified as an independent predictor of adverse cardiovascular outcome in HF patients (14, 31, 35, 36). In our cohort of iron-deficient HF patients we now observed that levels of MVs were considerably higher compared to healthy controls. In relation to other MV subpopulations, EMVs were the most drastically augmented population and displayed a more apoptotic phenotype, revealing an altered condition of the endothelium in HF patients. Notably, application of FCM resulted in a rapid, substantial, and prolonged shedding of EMVs into the circulation peaking at day one and normalizing after 14 days. Such augmentation of EMV levels was even more pronounced in HF patients with CKD, who seem to be at particular risk since a dysfunctional endothelium may represent a critical link accounting for the risk of both renal impairment and cardiovascular complications (32). Analysis of circulating MV subpopulations suggests that iron substitution dominantly affects the endothelium rather than circulating cells, as the change in MV levels was most accentuated for EMVs, moderately pronounced for PMVs and not observed for leukocyte- or erythrocyte-derived MVs. Of note, the potential of FCM to directly mediate the release of EMVs was corroborated by *in vitro* experiments demonstrating a dose- and time-dependent vesiculation when HUVECs were treated with pharmacologically relevant iron concentrations. The moderate and transient augmentation of PMV levels suggest an early but temporary activation of platelets in response to FCM administration. It remains to be elucidated, whether such temporary alteration in PMV levels may relevantly modify the procoagulatory and prothrombotic capacity and potential in HF patients.

Circulating EMVs display a dynamically controlled phenotypic heterogeneity which is thought to relate to the distinct pathophysiological process and context that triggers their release (29). Accordingly, the pattern of membrane proteins expressed by EMVs may reflect the homeostatic state of the endothelium and the vasculature. In line with this concept, clinical data revealed an association of specific circulating EMV phenotypes with diagnosis, disease severity, treatment, and prognosis of cardiovascular diseases (31, 37). We now performed temporal phenotypic profiling of EMVs to provide further insight into the nature of alteration of the endothelium following FCM application. The increase in EMVs expressing the inducible markers CD62E and CD54 indicates a swift and profound inflammatory activation of the endothelium just few hours after FCM application. In line with this notion, we observed in our *in vitro* experiments that FCM may indeed directly induce inflammatory activation of endothelial cells with increased expression of the adhesion molecules CD62E and CD106. The early rise in the CD62E+/CD31+ and CD54+/CD144+ EMV ratio emphasizes a striking phenotype shift of generated EMVs and confirms that shortly after FCM application endothelial activation is the dominant driver of increased EMV levels rather than endothelial apoptosis. At later stages, we observed a resolution of the enhanced activation state of the endothelium with CD62E+/CD31+ and CD54+/CD144+ ratio returning to baseline values, revealing reversibility of the phenotype shift and demonstrating that enhanced vascular activation is a temporary phenomenon following FCM application. We furthermore noted that at baseline and at a steady state ≥14 days after FCM exposure, EMVs from patients with HF displayed a generally lower CD62E+/CD31+ and CD54+/CD144+ ratio than EMVs from healthy subjects, indicating a higher degree of endothelial apoptosis in HF patients and supporting the concept of dysfunctional endothelium in HF patients. Altogether, temporal and phenotypic analysis of EMVs helps to unravel the pathophysiological condition of the vasculature and highlights that intravenous FCM application may temporarily aggravate the pre-existing disturbances of the endothelium present in HF patients.

MVs represent a system for efficient transfer of biological information and transduction of fundamental functions across a wide range of cell and tissue types (38). Therefore, circulating EMVs play not only a role as a marker of vascular health and potential predictor of cardiovascular events, but may act as a potent effector in various pathologies (15). To ensure homeostatic control under various settings, the composition and loading of MVs is finely tuned and responsive to changes in contextual and microenvironmental cues. According to this concept, the role of EMVs might change under pathophysiological conditions. Exemplarily, it has been shown that EMVs derived from glucose-treated endothelial cells are characterized by a reduced endothelial repair capacity *in vitro* and *in vivo*, substantiating the general notion of context-specific and stimulus-dependent function of MVs (39). In light of such variability of EMVs, data on their role has been rather conflicting with documentation of diverse and partly contradictory functions (18, 27, 40). On the one hand, studies demonstrated a potential of EMVs to support atherothrombosis and vascular inflammation as well as to promote oxidative stress (41). On the other hand, studies reported a vasculoprotective, pro-angiogenic, and regenerative role of EMVs and suggested that release of EMVs indicates the engagement of a defense and survival program that counteracts processes related to apoptosis and supports the maintenance of endothelial integrity (39, 42–44). The complexity and ambivalence of EMV functions emphasizes that further studies are needed to explore EMVs generated in response to supplemented iron beyond a surrogate parameter of endothelial activation and dysfunction. To assess their impact on vascular homeostasis and their potential to modify the progression of endothelial dysfunction and inflammation as well as to determine their clinical relevance in the setting of HF, decipherment of the specific functional capacity of EMVs induced by exposure to iron would help to address concerns regarding adverse vascular effects and safety of intravenous iron supplementation.

The mobilization, homing and recruitment of EPCs, programmed to accelerate vascular recovery, is part of the multifaceted endothelial response to injury (19). We now observed in our HF cohort that administration of FCM induced a two-staged reaction on the levels of circulating CD45lowCD34+KDR+ EPCs with an initial and rapid decrease, followed by substantial mobilization of EPCs peaking at day seven. A multimarker strategy with the combined detection of EMV with EPC levels may establish a more integrative marker of vascular health and may be useful to identify patients with progressing endothelial dysfunction due to an imbalance of endothelial injury and repair (15, 30). Our assessment of the EMV/EPC ratio with its biphasic sequence emphasizes that endothelial activation precedes accumulation of regenerative EPCs in the peripheral circulation and suggests that the initially disturbed vascular repair capacity becomes substantially engaged at later stages, putatively in an effort to address aggravated endothelial stress induced by FCM application. The reason for the temporally decreased EPC levels and decoupling from the development of EMV levels remains elusive, however it can be speculated that the stimulated endothelium with upregulated adhesion molecules may more abundantly capture EPCs from the circulatory pool before compensation by induced mobilization steps in and becomes relevant. Alternatively, apoptosis of EPCs may occur in response to FCM exposure. Interestingly, we detected relevantly lower levels and an alleviated mobilization of circulating EPCs in the CKD subgroup. Together with the identification of higher EMV levels, this resulted in a marked shift in the EMV/EPC ratio, confirming the general finding of a hampered vasculoprotective and regenerative capacity in the presence of CKD (45). Interestingly, we observed a continuous trend towards reduced levels of IL6 but not hsCRP in HF patients following FCM treatment (Mause unpublished data 2022, non-significant). Reduction of IL6 at stages with elevated EPC levels and enhanced repair capacity treatment may suggest attenuation of vascular inflammation days and weeks following treatment with FCM in HF patients.

Mobilization and vascular recruitment of EPCs is substantially governed by injury-induced expression of the chemokine CXCL12 (46, 47). Notably, our *in vitro* experiments now revealed that FCM may induce upregulation of endothelial CXCL12 expression, thus establishing a possible mechanistic link for the observed FCM-triggered mobilization of EPCs. Furthermore, MVs shedded by stressed and activated cells may also play an important role in such auto-regenerative processes due to their potential to convey and disseminate CXCL12 signals, transfer proangiogenic factors and the CXCL12 receptor CXCR4 to neighboring cells and enhance the regenerative potential of EPCs (24, 38, 46, 48). Indeed, it has been shown that EMVs derived from apoptotic endothelial cells promote endothelial proliferation and mediate anti-apoptotic effects *via* CXCL12 and that systemic treatment of mice with EMVs accelerated reendothelialization and reduced neointima formation after vascular injury (43, 49).

In summary, our study shows early but temporary endothelial dysfunction related to intravenous FCM administration in HF patients, but also indicates subsequent engagement of an auto-regulatory feedback loop with increased mobilization of EPCs potentially supporting endothelial recovery and reconstitution of vascular health. It remains to be elucidated, whether EMVs generated upon exposure to FCM are solely a surrogate parameter of endothelial disturbances, further promote or aggravate endothelial dysfunction in HF patients or are actively involved in the initiation and propagation of a defense program contributing to alleviated apoptotic processes and an enforced repair capacity with increasingly mobilized EPCs. Despite the overall clinical benefit of intravenous FCM supplementation in HF patients with iron deficiency, our study emphasizes the rationale and plausibility to avoid iron overcorrection as well as overloading and warrants the need to monitor potential vascular adverse effects of intravenous iron supplementation, in particular in scenarios with repetitive FCM administration due to persistent iron deficiency and in high-risk populations such as CKD patients with an impaired endothelial repair capacity.

Limitations of our study must be acknowledged. First, the etiology of HF is heterogeneous in HF patients seen in daily clinical practice as well as in our cohort. Moreover, HF encompasses a wide range of structural and clinical phenotypes and comprise different entities, such as HF with preserved vs. reduced ejection fraction. Various pathologies underlying HF may differentially affect not only the inflammatory state of the vasculature and oxidative stress level, but also endothelial homeostasis, (preexisting) endothelial dysfunction and intrinsic vasoregenerative capacity. Consequently, levels and characteristics of circulating EMVs and EPCs may relevantly vary between different HF etiologies and entities. Second, the sample size of our study is limited and too low to allow discriminative analysis of various etiological HF subgroups, emphasizing the need for larger studies to consider and address the heterogeneity and complexity of HF pathologies. Third, limitations in the clinically applicable assessment of circulating MVs and EPCs in blood samples remain despite ongoing improvement and refinement of flow cytometric analysis as well as substantial progress in the collective knowledge regarding preanalytical and analytical handling (50). Further research into the effects of preanalytical variables, optimization of MV sizing and counting methods, and minimization of artefact detection are necessary together with general optimization in standardization of MV analysis.

## Data availability statement

The original contributions presented in the study are included in the article/supplementary material. Further inquiries can be directed to the corresponding author.

## Ethics statement

The studies involving human participants were reviewed and approved by Ethic committee of RWTH Aachen University (Aachen, Germany). The patients/participants provided their written informed consent to participate in this study.

## Author contributions

SM, VB and RS conceived and designed the research. SM and HYL performed the experiments. SM and MB analyzed data and interpreted results. VB and RS collected material. SM prepared figures. SM drafted the manuscript. SM, MB, HYL, FV, VB, and RS edited and revised manuscript. All authors contributed to the article and approved the submitted version.
